# Experimental Characterization of a Flexible Thermal Slip Sensor

**DOI:** 10.3390/s121115267

**Published:** 2012-11-08

**Authors:** Maria Teresa Francomano, Dino Accoto, Eugenio Guglielmelli

**Affiliations:** Laboratory of Biomedical Robotics and Biomicrosystems, Università Campus Bio-Medico di Roma, Via Alvaro del Portillo 21, Roma 00128, Italy; E-Mails: d.accoto@unicampus.it (D.A.); e.guglielmelli@unicampus.it (E.G.)

**Keywords:** slip detection, flexible sensors, tactile sensors

## Abstract

Tactile sensors are needed for effectively controlling the interaction between a robotic hand and the environment, e.g., during manipulation of objects, or for the tactile exploration of unstructured environments, especially when other sensing modalities, such as vision or audition, become ineffective. In the case of hand prostheses, mainly intended for dexterous manipulation of daily living objects, the possibility of quickly detecting slip occurrence, thus avoiding inadvertent falling of the objects, is prodromal to any manipulation task. In this paper we report on a slip sensor with no-moving parts, based on thermo-electrical phenomena, fabricated on a flexible substrate and suitable for integration on curved surfaces, such as robotic finger pads. Experiments performed using a custom made test bench, which is capable of generating controlled slip velocities, show that the sensor detects slip events in less than 50 ms. This response time is short enough for enabling future applications in the field of hand prosthetics.

## 1. Introduction

Current efforts in the field of robotic upper limb prosthetics are focused on the development of devices able to restore compromised body functions, with the final aim of achieving performances comparable to those of unimpaired people [1].

Slip detection is crucial for performing effective manipulation tasks. In fact, during the manipulation of delicate or fragile objects, it is important to reduce interaction forces to their lowest effective value, in order to avoid damaging the object, while still guaranteeing a stable grasp. Slip perception in humans is based on a concurrent involvement of different skin mechanoreceptors [2], as well as of predictive internal models [3]. The absence of a unique and specific receptor for slip detection is probably the main reason why mimicking this human sensing capability is still complex and why over the years several different approaches for the development of slip sensors have been pursued, based on the observation of a variety of physical phenomena and quantities including: object displacement [4,5], microvibrations [6–9], normal and shear forces [10–13], thermal fluxes [14]. Similarly, several trans-duction mechanisms (e.g., piezoresistive [15], piezoelectric [16–19], capacitive [20], optical [5], magnetic [21], thermal [14]) as well as fabrication technologies (e.g., thick-film [16], soft/flexible materials [20,22], MEMS [5,23]) have been explored.

However, despite the fact that the idea of embedding slip sensors in prosthetic hands dates back to the 1980s [24], most existing slip sensors are still: (i) affected by external disturbances; (ii) technologically demanding; (iii) expensive; or (iv) not embeddable on top of robotic finger pads.

The sensor we describe in this paper has no moving parts and exploits a thermo-electrical transduction principle, introduced in [14] and briefly outlined in Section 2. Since the sensor does not rely on the detection of vibrations, mechanical noise, e.g., produced by motors, does not affect its performance. Moreover, it is equally effective on rough and smooth objects.

Compared to the prototype in [14], the sensor described in this paper has been microfabricated on a flexible substrate and the contact layer has been removed in order to improve the heat flux exchanged between the microheater and the object surface.

The design and microfabrication are reported in Section 3, while the characterization set-up and the detection strategy are detailed in Section 4. Experimental results, which extend and detail the preliminary ones reported in [25], are discussed in Section 5. Finally, Section 6 is devoted to Conclusions and Future Work.

## 2. Working Principle

**Figure 1.**
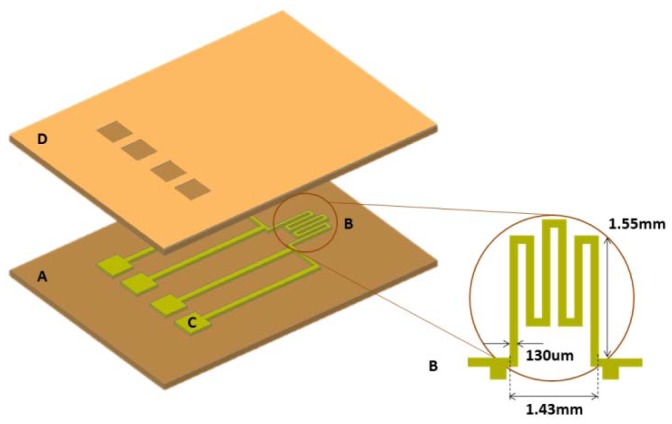
A 3D schematic of the sensor; (**A**) polyimide substrate; (**B**) microheater; (**C**) pads; (**D**) polyimide covering layer.

The sensing element is a patterned thin metal film with a positive thermal coefficient (α), working as a microheater (Figure 1). The metal film is warmed up by the Joule effect. By measuring its electrical resistance (R), direct information on its temperature (T) can be retrieved without the need for a dedicated temperature sensor, by inverting the following equation (where R_0_ is the resistance at the reference temperature, T_0_):

(1) $$\text{R}(\text{T})={\text{R}}_{0}+[\alpha (\text{T}-{\text{T}}_{0})]$$

The microheater is kept at a constant temperature, which must be above the object's temperature, by a dedicated control, described in Section 4.2. When the sensor is in contact with an object with zero relative speed (v = 0, no slip condition), only heat conduction occurs, according to Fourier's law:

(2) $$\partial \text{T}/\partial \text{t}=\text{k}\nabla^{2}\text{T}+\text{q}(\text{x})$$

where q(x) is the power generation term (Joule effect), and k is the thermal diffusivity [m^2^/s], defined by:

(3) $$\text{K}=\text{K}/\left({\text{C}}_{\text{p}}\rho \right)$$

In (3) K, C_p_ and ρ are the thermal conductivity [W/m K], the specific heat [J/kg K] and the density [kg/m^3^] of the medium through which heat conduction occurs, respectively. If a slip event occurs (v ≠ 0), a convective heat transfer term (**v**·grad T) adds to the left member of (2):

(4) $$\partial \text{T}/\partial \text{t}+\text{v}\cdot \text{gradT}=\text{k}\nabla^{2}\text{T}+\text{q}(\text{x})$$

The additional convective term requires a higher current to keep the microheater at the desired temperature. The slip detection strategy consists in firing a signal whenever such current overcomes a threshold.

It is therefore evident that the proposed detection strategy is inherently incapable of detecting slip incipience: a finite slip velocity is indeed necessary to build up a convective heat transfer term, responsible for the increase of the absorbed current. Nonetheless, it is possible to demonstrate that the sensitivity of the sensor to slip velocity is maximum for very small velocities, because the additional convective term quickly increases when slip velocity goes from zero to a given finite value.

Let's schematize the sensor as a circular isothermal sensing area sandwiched between a substrate and a protective layer (Figure 1). Because of their small masses, the thermal capacitances of the layers can be neglected compared to the thermal capacitance of the touched object. This allows the development of a purely resistive thermal model. If the thickness of the protective layer is very small compared to the transversal linear dimension of the microheater, a 1-D model can be used. Let Q_c_ be the thermal flux due to convection. Q_c_ is a function of a number of parameters: ν, slip speed; ΔT, temperature difference between the protective layer and the object; K, object thermal conductivity; S, contact surface; ρ, object density; C_p_, specific heat of the object. All the above quantities can be expressed in terms of four fundamental units: Mass, Length, Time and Temperature. Therefore, according to Buckingham's theorem, the thermal problem:

(5) $${\text{Q}}_{\text{c}}={\text{Q}}_{\text{c}}(\text{v},\;\Delta \text{T},\text{K},\text{S},\rho ,{\text{C}}_{\text{p}})$$

can be expressed in an equivalent form using only three dimensionless groups, which can be easily calculated. Moreover, taking into account that a quadratic relation exists between slip velocity and thermal power [26] and that heat flux must be directly proportional to ΔT, one gets:

(6) $${\text{Q}}_{\text{c}}=\text{A}\;{\text{S}}^{3/4}\;\Delta \text{T}{\left(\text{v}\;\text{K}\rho {\text{C}}_{\text{p}}\right)}^{1/2}$$

where A is a dimensionless constant. By using the thermal diffusivity (k), (6) can be also written as:

(7) $${\text{Q}}_{\text{c}}=\text{A}\;{\text{S}}^{3/4}\;\text{K}\;\Delta \text{T}{\left(\text{v}/\text{k}\right)}^{1/2}$$

The sensitivity of Q_c_ to slip velocity (∂Q_c_/∂v)) is singular when v = 0:

(8) $$\partial {\text{Q}}_{\text{c}}/\partial \text{v}=[\text{A}\;{\text{S}}^{3/4}\;\rho \;{\text{C}}_{\text{p}}\;\Delta \text{T}]/[2{\left(\text{v}/\text{k}\right)}^{1/2}]$$

In conclusion, although the sensor is not capable of detecting incipient slips, its responsiveness at low slip speeds is just limited by the resolution of the readout electronics used to calculate T through the measure of R, according to (1), being the additional convective term very sensitive to slip speed during the very initial instants of slip occurrence.

## 3. Fabrication and Thermal Characterization

The multilayered structure shown in Figure 1 includes a polyimide substrate, a thin metal film layer and a protective polyimide coat. The metal film is sandwiched between the two polyimide layers, which have the same thickness, thus laying on the neutral plane of the structure. This configuration preserves the metal from possible structural failures caused by tensile stresses that may arise during bending.

The active area of the sensor is an Au microheater, 25 nm thick, shaped as a serpentine, 130 μm wide, with a total length of 15.5 mm and a surface area of 2 mm^2^ (Figure 1). The connection between the microheater and the readout electronics occurs through two pairs of electric contacts: one pair is dedicated to current supply, the other one is used to read the electric resistance of the heating element. The dimensions of the electrical connections have been chosen in order to make the voltage drop across them negligible, compared to the voltage drop across the microheater.

The microfabrication of the sensors includes standard photolithographic and lift-off processes, the main steps being as follows:

### Substrate

15 μm of polyimide (Duramide 115A) has been spun on a sacrificial 4″ silicon wafer. A soft (at 100 °C for 120 s) and hard bake (at 350 °C for 1 h 30′) process have also been performed.

### Metal Layer

A lift-off process has been performed using a negative photoresist (ma-N 400 microresist), patterned by means of a photolithographic process. In order to allow the adhesion between the Au and the polyimide layers, a multilayer metal film, Cr/Au/Cr (thickness: 2.5 nm/25 nm/2 nm) has been evaporated.

### Protective layer

15 μm of photodefinable polyimide has been patterned in order to cover the metal layer, while keeping the pads open for electrical connections. Finally, in order to avoid pad oxidation, the 2 nm Cr layer has been etched.

**Figure 2.**
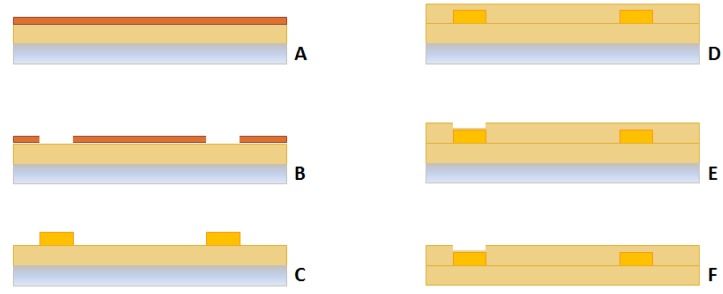
Microfabrication process. (**A**) Deposition of 15 μm of polyimide on a Si wafer; spin coating of a negative resist; (**B**) patterning of the negative resist; (**C**) metals deposition; (**D**) 15 μm polyimide deposition; (**E**) pad opening (photolithography); (**F**) Si sacrificial layer removed by TMAH attack.

**Figure 3.**
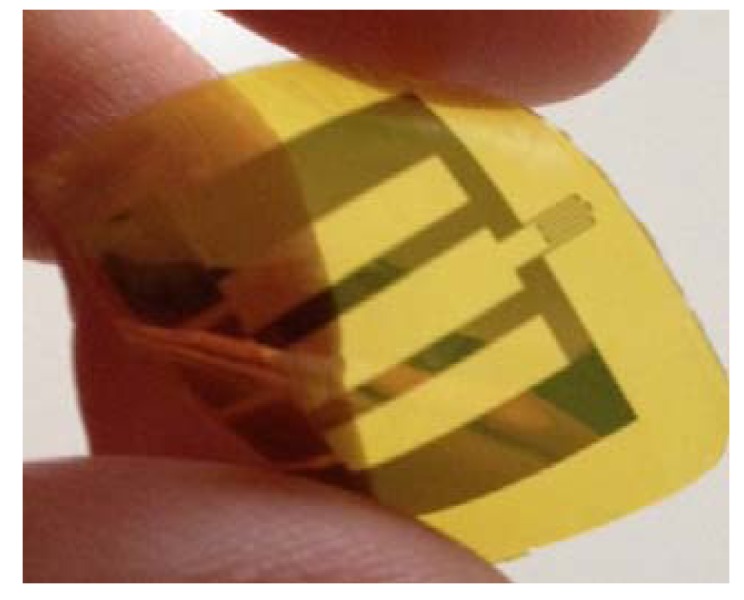
Picture of a bent slip sensor.

The complete structure (polyimide/metals/polyimide) has been removed from the Si sacrificial substrate by using a tetramethylammonium hydroxide (TMAH) attack. Figure 2 shows a schematic of the microfabrication process. A picture of the actual sensor is shown in Figure 3.

**Figure 4.**
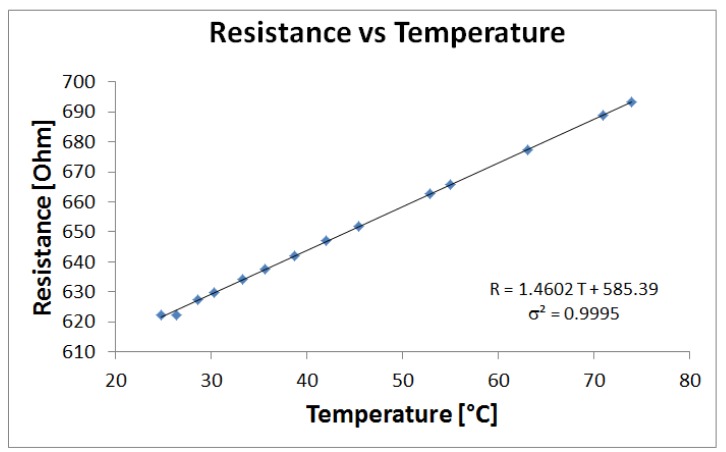
Thermal characterization of the sensor.

The temperature-resistance characteristic of the sensor has been obtained using a Type-K thermocouple (Model 80BK-A, by Fluke) and a OHM meter (Model 189, by Fluke). As shown in Figure 4, data can be fitted linearly (σ^2^ = 0.9995): R = 1.4602 T + 585.39, where the resistance R is in [Ω] and the temperature in [°C].

The maximum temperature which the sensor can safely withstand has been determined experimentally to be around 75 °C. In any case, the maximum working temperature of the sensor is about 40 °C, which is a temperature higher than that of commonly handled objects, but low enough to avoid damages in case of prolonged contact with objects or human skin.

Since the temperature of the microheater increases with the increase of the supplied current, it is necessary to keep this current below a safety threshold. When a constant current is provided, the temperature reached by the microheater depends on all those factors affecting the boundary conditions of the thermal problem, including the mounting/packaging of the sensor and the thermal and geometric properties of the touched objects. A simplifying and cautionary approach for evaluating the maximum current, which can safely be fed to the sensor, consists in limiting its heat exchanges with the environment, e.g., by having it suspended in air.

**Figure 5.**
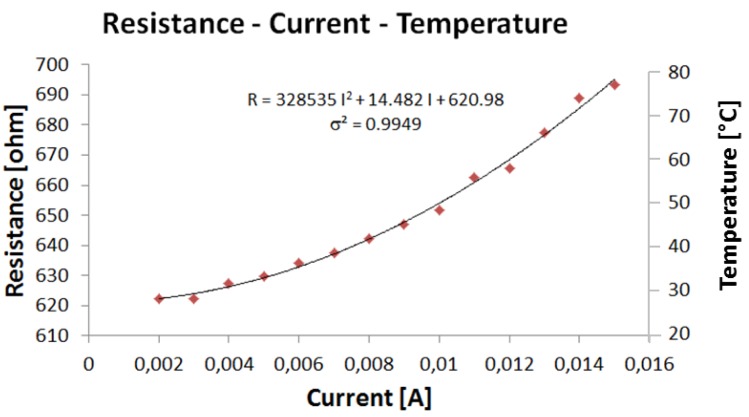
Experimental relation between the current supplied to the sensing element and its resistance variations when the sensor is suspended in air.

In this configuration, different current values, ranging from 1 mA to 15 mA have been provided to the microheater. Figure 5 shows the data obtained, which can be fitted by a quadratic relation (σ^2^ = 0.9949): R = 328,535 I^2^ + 14.482 I + 620.98, where the resistance R is in [Ω] and the current in [A].

## 4. Experimental Section

### 4.1. Experimental Set-up

**Figure 6.**
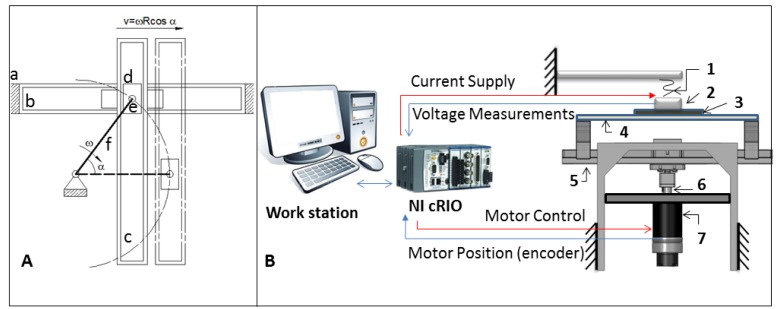
(**A**) Schematic of the Scotch-Yoke mechanism; (**a**) Frame; (**b**) Horizontal linear guide; (**c**) Vertical linear guide; (**d**) Slider; (**e**) Pivot; (**f**) Crank. (**B**) The whole experimental set-up, including (i) a work station, running Labview RT (by National Instruments); (ii) a programmable automation controller (NI CompactRIO, by National Instruments); and (iii) the custom made mechanical set-up. (**1**) Elastic element; (**2**) Thermal slip sensor; (**3**) Slipping bar; (**4**) Plate; (**5**) Linear guide; (**6**) Crankshaft; (**7**) DC Motor.

A custom set-up, capable of providing controlled slip speeds, has been developed. A Scotch-Yoke mechanism in the system (Figure 6A), named hereafter “slip generator”, has been assembled for converting the constant rotary motion of a crank into the linear harmonic oscillation of slipping bars. The slip sensor has been coupled to a fixed frame through an elastic element (Figure 6B), which compensates for possible geometric irregularities of the touched surface. The crank is moved by a DC gearmotor (Maxon Motor EC 45 Flat 50 W; reduction ratio: 26:1) with an embedded incremental encoder (2,000 pulses/turn). Measurements of the microheater resistance and the motor position have been performed using two DAQ cards, the NI9205 and the NI9401, respectively, while current has been supplied by another DAQ card, the NI9265. All the DAQ cards (by National Instruments) have been connected to a programmable automation controller (NI CompactRIO, by National Instruments).

Both temperature control and motor rotation control have been implemented on a PC, using LabviewRT (by National Instruments). The whole experimental set-up is shown in Figure 6B. Bars of different materials (polyvinyl chloride–PVC, polytetrafluoroethylene–Teflon, and pinewood) with a rectangular cross-section (25 mm × 70 mm; thickness: 3 mm) and similar surface roughness (2.5/4 μm Ra) have been fixed on the frame, with one side in contact with the sensor.

### 4.2. Detection Strategy

In order to keep the microheater temperature at a constant value, a two-state bang-bang control has been implemented, with only two current intensities supplied to the microheater: I_L_ and I_H_ (I_L_ < I_H_).

Let R_T_ be the target resistance (higher than the one measured across the sensing element at room temperature), and T_T_ the corresponding temperature of the microheater. Once T_T_ is reached, the voltage drop across the microheater can assume two reference values: V_L_ = R_T_ I_L_ or V_H_ = R_T_ I_H_.

According to (1), when a constant current is supplied, changes in the microheater temperature cause proportional variations to V_L_ and V_H_. Therefore, after each sampling period, T_s_, the voltage drop across the microheater is compared to the reference voltage (*i.e.*, to V_L_ if the supplied current is I_L_; to V_H_ if the supplied current is I_H_).

**Figure 7.**
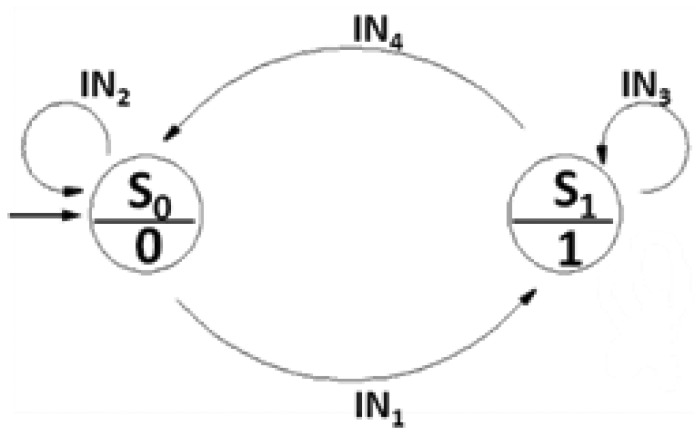
States diagram.

Let's define S_0_ and S_1_ as the states when I_L_ and I_H_ are supplied, respectively. Starting from the initial state S_0_, if the measured voltage is lower than the reference voltage V_L_ (*i.e.*, R < R_T_), it is necessary to heat up the microheater. Therefore a transition from S_0_ to the S_1_ is required (S_0_→S_1_). Otherwise, (*i.e.*, R > R_T_), no transition occurs. Once in S_1_, if the measured voltage is lower than the reference voltage V_H_, it is necessary to keep heating the sensing element, and no state transition occurs. On the contrary, if the measured voltage overcomes V_H_, it is necessary to cool down the microheater by switching to I_L_ (S_1_→S_0_). All the possible state transitions are summarized in Table 1 and graphically shown in Figure 7. By using the symbols in Table 2, the truth table associated to the state diagram can be built (Table 3).

**Figure 8.**
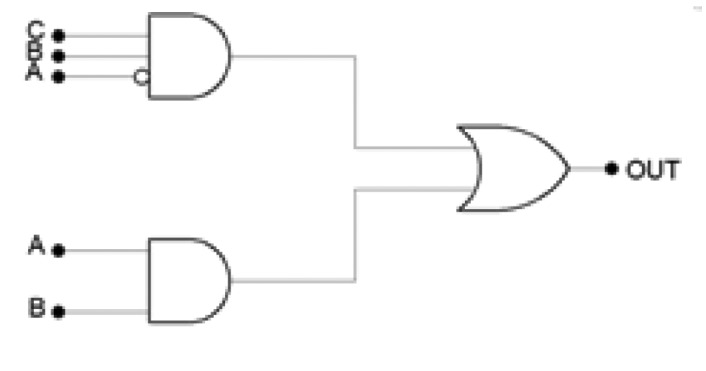
Simplified logical circuit, implementing the bang-bang temperature control.

The corresponding control algorithm can be implemented using a logical multi-level network (Figure 8), simplified using Karnaugh's maps [27].

Binary values can be used to represent the two possible values of the supplied currents: “1” stands for I_H_ and “0” for I_L_. The values of supplied currents are stored in a binary string (**I**). During an experimental session a value (“1” or “0”) is appended to **I** every T_s_ seconds.

The analysis of **I** is sufficient to detect slip: when slip occurs, the increase of the average current provided to the microheater corresponds to an increase in the average length of the substrings of **I** made of consecutive “1”s. Alternative indices, able to detect a (statistically) significant increase of the number of “1”s in any substring A of **I** containing a Δ-uple of binary values, can be used. In [25] we proposed the use of the coefficient of variation, defined as:

(9) CV=σ/μ

where σ and μ respectively are the standard deviation and the mean of the values in A. It is easy to show that, for a binary string, σ is not independent from μ, since: σ = (μ − μ^2^)^1/2^.

Therefore, the coefficient of variation can be equivalently written as:

(10) $$\text{CV}={\left[(1/\mu )-1\right]}^{1/2}$$

If n_1_ is the number of “1”s in A, μ = n_1_/Δ, and:

(11) $$\text{CV}={\left[(\Delta /{\text{n}}_{1})-1\right]}^{1/2}$$

CV approaches 0 (its minimum) when μ tends to 1 (its maximum), *i.e.*, when n_1_→Δ. Let CV_T_ be a threshold value, defined experimentally during tests performed in pure conduction conditions (*i.e.*, no slip). The sensor would fire a slip signal when:

(12) $$\text{CV}<{\text{CV}}_{\text{T}}$$

The value of CV_T_ can be set by performing tests in pure conduction conditions (no slip). Once the electrical parameters are fixed (*i.e.*, I_L_, I_H_, R_T_), a binary string **I** (length: N) is stored during each test. Fixing the length Δ of the substrings (Δ was set to 10 in our experiments), a coefficient of variation is calculated for each of the N-Δ substrings [**I**_k_-Δ, **I**_k_], with k ∈ (Δ + 1,N). CV_T_ is obtained as the mean of the N-Δ coefficients of variations previously calculated.

During slip tests, every T_s_ seconds, the current value of CV, CV_t_, is calculated considering the last Δ values stored in **I**. The one-tailed Student's t-test is implemented to evaluate if CV_T_ is significantly higher than CV_t_ (12). If so, a slip signal is fired.

## 5. Results and Discussion

Bars of different materials (*i.e.*, PVC, Teflon and pinewood) have been used. The bars have been fixed onto the slip generator, with one side in contact with the sensor (Figure 6B). Table 4 summarizes the values of the experimental parameters, while the thermal properties of the selected materials are reported in Table 5.

### 5.1. Power Dissipation

**Figure 9.**
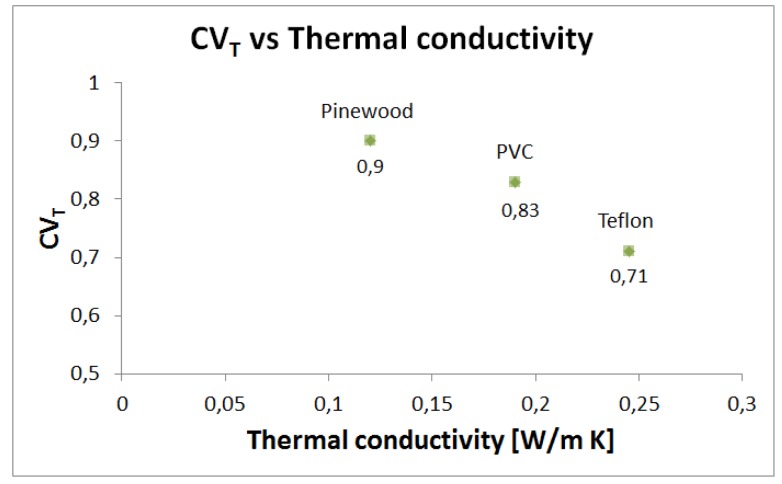
CV_T_ *vs*. Thermal conductivity for the tested materials.

**Figure 10.**
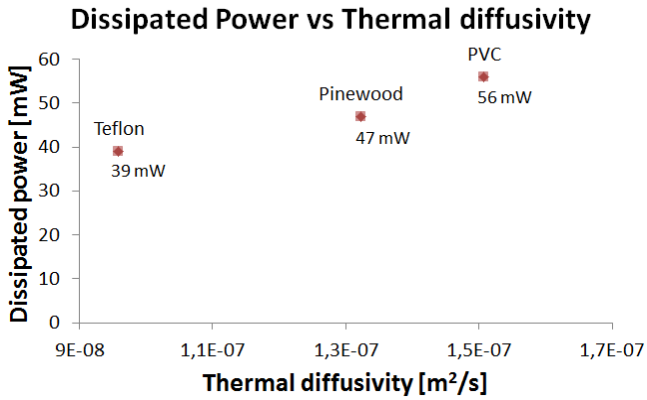
Dissipated power *vs.* thermal diffusivity for the tested materials.

In first instance, pure conduction tests have been performed on the three materials (Table 5) to set the values of CV_T_, according to the methodology described in Section 4.2. During the same tests, the average dissipated power over a time window of 60 s, in steady conditions, with I_L_ = 1 mA, I_H_ = 13 mA and R_T_ = 616 Ω, has also been measured. Figure 9 shows that the CV_T_ decreases with thermal conductivity. Indeed, by keeping the electrical parameters (*i.e.*, I_L_, I_H_ and R_T_) constant, when the sensor is in contact with an object with a higher thermal conductivity, a higher heat exchange occurs, which requires more power for maintaining the constant temperature (Figure 10).

The dissipated power, *i.e.*, the power absorbed in absence of slip, is significantly lower than that reported in [14], thanks to the design optimization [26] and the removal of the contact layer.

### 5.2. Response Time

**Figure 11.**
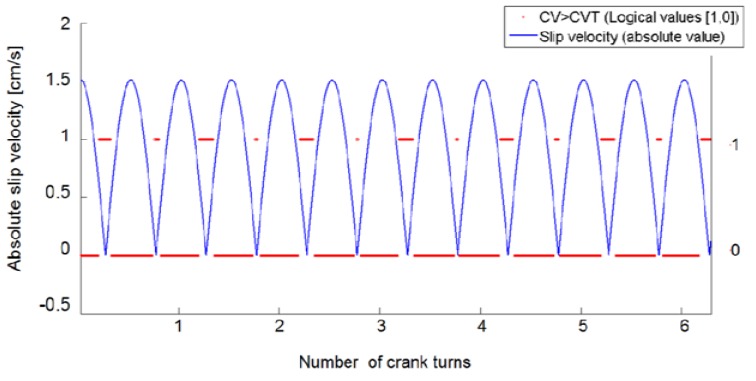
CV *vs*. numer of crank turns, during a slip test.

When the motor rotates at constant speed (ω = 150 rpm, Table 4), slip velocity varies in time as described by the solid blue line shown in Figure 11. The peak speed of the bars during their harmonic oscillations (peak slip velocities), calculated taking into account the reduction ratio of the gear head (τ) and the length of the crank (R_m_), is 1.5 cm/s.

**Figure 12.**
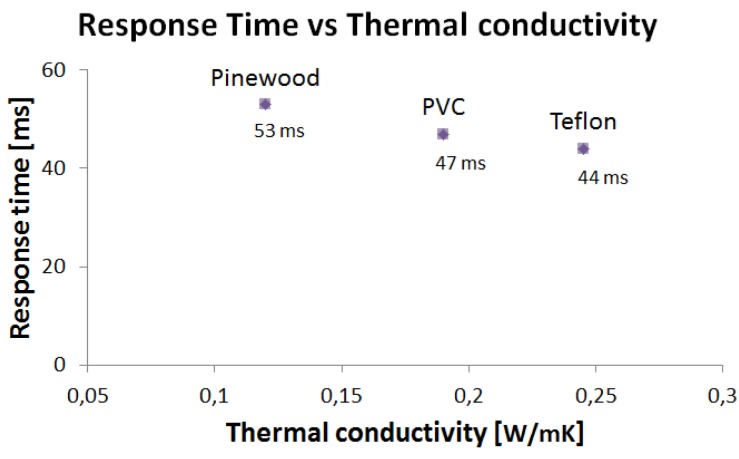
Response time of the thermal slip sensor.

The string **I** has been used to evaluate CV_k_ over the substrings [**I**_k_-Δ, **I**_k_]. Figure 11 shows an example of data obtained using the PVC bar. The (absolute) value of slip speed (expressed in [cm/s]) is reported as a function of the number of crank turns (solid line in Figure 12), while the dashed horizontal lines stand for the logical values (1,0): “1” refers to the condition CV ≥ CV_T_ (no slip), while “0” to the condition CV < CV_T_ (slip detected). A slip signal is fired at instant kT_s_ provided that CV_k_ < CV_T_, according to the procedure described in Section 4.2.2. If the bar was at rest at instant t, the response time can be calculated as kT_s_ − t.

As shown in Figure 12, the response time decreases with thermal conductivity. In particular, an almost 100% variation of the thermal conductivity corresponds to a variation of 17% of the response time. In the case of materials with very high thermal conductivities, such as metals (iron, for example, has a thermal conductivity three orders of magnitude higher than that of Teflon), a dramatic decrease of the response time is expected, which would require the development of an adequately fast readout electronics.

For all the materials tested, the measured response times are significantly shorter than those of humans [2]. Short response times allow enough computation time to be allotted to the system controlling the grasping, still keeping the overall responsiveness of the artificial system in line with that of the human sensory-motor system.

## 6. Conclusions and Future Work

A thermal slip sensor with no-moving parts has been developed on a flexible substrate, in order to allow its mounting on curved surfaces, such as robotic finger pads. The sensing element, an Au microheater, has been sandwiched between two 15 μm thick polyimide layers. This configuration implies that the metal, lying on the neutral plane of the structure, is not affected by any compressive/tensile stresses during sensor bending.

The sensor has been functionally characterized. To this aim a dedicated control has been implemented for keeping the sensor at a constant temperature, and an experimental mechanical set-up has been developed in order to perform slip tests on three different and frequently used materials.

Experiments show the sensor capability in detecting slip events in about 40–55 ms, depending on the slipping material. On-going work is focused on implementing an adaptive control able to autonomously set the threshold value of slip occurrence (CV_T_) as well as on characterizing the sensor capability in discriminating initial contacts from slip events. Moreover, the readout electronics and the control will be further optimized and improved in order to be able to test the sensor with bars of several materials, covering a wider range of thermal conductivity values. Moreover, in order to improve the spatial acuity, as well as to allow the detection of slip direction, ongoing works focus on the development and characterization of a 2 × 1 cm^2^ array, embedding 24 thermal slip sensing units, arranged in a chessboard-like configuration.

Future work will be devoted to investigate possible performance limits due, for example, to the thickness of the touched object (e.g., thin paper sheets). Finally, considering that the response time is influenced by the thermal conductivity of the object, an extensive characterization of the sensor will be performed in order to verify its possible capability of discriminating the material it is in contact with. It is expected that the slip of objects with comparable thermal properties will induce comparable response times, but further optimizations of the sensing elements should overcome this issue.

Finally, in order to test the effectiveness of the sensors in a real scenario, future work includes the sensors integration on a robotic fingertip and the development of a manipulation control scheme, integrating the sensor signals.

## Figures and Tables

**Table 1.** Table of States.

**Input**	**Description**	**Transition**
IN_1_	V < V_L_	S_0_→S_1_
IN_2_	V ≥ V_L_	S_0_→S_0_
IN_3_	V < V_H_	S_1_→S_1_
IN_4_	V ≥ V_H_	S_1_→S_0_

**Table 2.** Logical conditions.

**Symbol**	**Condition**	**Logical Value**
A	V < V_L_	1
B	V < V_H_	1
C	I = I_H_	1

**Table 3.** Truth table.

**A**	**B**	**C**	**Output**
0	0	0	0
0	0	1	0
0	1	0	0
0	1	1	1
1	0	0	N.V. *
1	0	1	N.V. *
1	1	0	1
1	1	1	1

* N.V.: Not verifiable.

**Table 4.** Parameters set during trials.

**Symbol**	**Description**	**Value [ref.]**
I_L_	Current (low)	1 mA
I_H_	Current (high)	13 mA
R_0_	Microsensor resistance at room temperature	614 Ω
R_T_	Microsensor target resistance	616 Ω
α	Au thermal coefficient	0.0034 °C^−1^[28]
ω	Motor velocity	150 rpm
τ	Reduction ratio	26:1
T_S_	Sampling time	1 ms
R_m_	Crank radius	25 mm

**Table 5.** Thermal properties of bar materials.

**Material [ref.]**	**Thermal conductivity at 25°C, k [W/m K]**	**Specific heat, cp [J/kg K]**	**Density, ρ [kg/m^3^]**
PVC [29]	0.19	900	1,400
Teflon [30]	0.245	1,172	2,180
Pinewood [30]	0.12	1,674	540
